# Prevalence of substance use among college students in Eldoret, western Kenya

**DOI:** 10.1186/1471-244X-11-34

**Published:** 2011-02-28

**Authors:** Lukoye Atwoli, Prisca A Mungla, Moses N Ndung'u, Kiende C Kinoti, Evans M Ogot

**Affiliations:** 1 Department of Mental Health, Moi University School of Medicine, PO Box 4606, Eldoret 30100, Kenya; 2 Moi University School of Medicine, PO Box 4606, Eldoret 30100, Kenya

## Abstract

**Background:**

Substance use among college and university students predicts substance related problems in later life. Few studies on this phenomenon have been carried out in low income countries, and most focus on primary and secondary school students. This study therefore aimed to establish the prevalence and factors associated with drug use among university and college students in a low income country.

**Methods:**

*Design: *A descriptive cross-sectional survey using the Self-Administered WHO Model Core Questionnaire to collect information on use of various drugs among students in colleges and university campuses within Eldoret Municipality in Western Kenya. *Setting: *Four tertiary learning institutions in Eldoret Municipality were randomly selected for inclusion in the study- three tertiary level non-university institutions and one university campus. *Subjects: *Five hundred students who gave consent to participate in the study, 125 from each of the four participating institutions. The mean age was 22.9 years (18-32, s.d. 2.5), and males made up 52.2% of the sample.

**Results:**

Lifetime prevalence rate of any substance use was 69.8%, and none of the socio-demographic factors was significantly associated with this. Lifetime prevalence rate of alcohol use was 51.9%, and 97.6% of alcohol users had consumed alcohol in the week prior to the study. The prevalence rate of cigarette use was 42.8%, with males having statistically significantly higher rates than females (p < 0.05). Other substances used were cannabis (2%) and cocaine (0.6%). Among those who admitted to using substances, 75.1% were introduced by a friend while 23.5% were introduced by a relative other than a member of the nuclear family. Majority of those using substances wanted to relax (62.2%) or relieve stress (60.8%). Problems associated with alcohol use included quarrelling and fights, loss and damage to property, problems with parents, medical problems and unplanned unprotected sex.

**Conclusion:**

The prevalence of substance use among college and university students in Eldoret is high and causes significant physical and psychosocial problems in this population. A large proportion of those using alcohol reported serious adverse effects, raising the necessity of targeted interventions to reduce the risk of subsequent substance dependence and other deleterious consequences.

## Background

Substance use among college and university students remains an important area of research due to the implications of early substance dependence on the future of the youth. Prior studies from various settings indicate relatively high rates of alcohol and other substance use among high school students and those in higher educational institutions [1-7]. Among the few studies from universities and colleges in Kenya, Odek-Ogunde et al reported high rates of substance use among students at a Kenyan private university, with rates as high as 84% for alcohol use and 54.7% for tobacco use [5].

A majority of the studies from this region focus on primary and secondary schools, and little information is available from college and university populations [2-4,8]. Most of these studies still find high rates of substance use among school-children, suggesting that the rates would continue to rise among students in institutions of higher learning.

For instance, Kuria [2] found alcohol use prevalence rates of upto 15% among secondary school students, while Kwamanga et al [3] found lifetime cigarette smoking rates of 32%. In a study among primary school pupils in Kenya, Ogwell et al [8] found a lifetime cigarette smoking rate of 31%. Among the very few similar studies from African countries other than Kenya, a South African study found an alcohol use prevalence rate of 39.1% and a cigarette use prevalence rate of 10.6% among high school adolescents [4]. Other drugs that are commonly used in these settings include cannabis, inhalants, tranquilisers, heroin and cocaine, among others [2,5].

The importance of these findings lies in the fact that early exposure to substance use often predicts future substance use and other psychiatric disorders [9,10]. Rohde and colleagues demonstrated that adolescent substance use disorder is associated with numerous functioning difficulties at age 30, some of which appear to be related to recurrent substance use disorder, co-morbid adolescent disorders, or functioning problems already evident in adolescence [11].

Some of the most studied risk factors for substance use among adolescents and young adults include low grade point average, lack of religiosity, early alcohol use, low self-esteem, psychopathology, poor relationship with parents, lack of social conformity (deviance), sensation seeking, perceived peer drug use, and perceived adult drug use [12]. No systematic study has been attempted to validate these factors in this region, and these factors are often cited as having universal application.

The paucity of data on substance use among college and university students in Kenya and other low income countries has serious implications on the success of any interventions aimed at reducing this problem. The importance of documenting this problem cannot therefore be overstated.

In this study, the main objective was to determine the prevalence and factors associated with substance use among students in tertiary institutions in Eldoret Municipality in Kenya, as well as to document any effects attributed to the use of various substances.

## Methods

### Site

The study was carried out at four tertiary institutions of learning within Eldoret Municipality in the then Uasin Gishu District, Kenya. They consisted of one privately-run diploma level college, a government-run Technical Training Institute, a government-run polytechnic and the Law campus of Moi University. At the time of the study, the Education office in the municipality had 12 registered tertiary institutions of learning, including 4 campuses of Moi University, the government-run polytechnic, the government-run technical training institute and 6 private colleges offering various certificate, diploma and higher diploma courses. Information about the student populations in these institutions is not available in the public domain, so it was not possible to compare them for various socio-demographic variables before selection for inclusion into the study. Four of these institutions with a total student population of 11,304 were selected through simple random sampling for participation in this study. Using Fisher's formula with an expected substance use prevalence rate of 84% (based on alcohol use prevalence rate from the study by Odek-Ogunde et al) and a confidence interval of 95%, a minimum sample size of 206 was calculated. However, we decided to recruit a higher total sample of 500 students in order to be able to make meaningful analyses, and a sample of 125 students per institution was allocated for selection through stratified sampling.

### Participants

Five hundred students were selected from the four institutions through a process of stratified sampling, with the sample allocation being proportionate to the number of students in each class in an institution. Within each class, simple random sampling was used to select the participants based on the student registration numbers obtained from the institutions' heads.

### Design

The study used a cross-sectional descriptive survey design involving the administration of the World Health Organisation self-administered Model Core Questionnaire to collect information on the use of various drugs including alcohol, tobacco, stimulants, marijuana, cocaine and heroin, among others [13]. The WHO model core questionnaire has been standardized and used elsewhere in Africa to collect data on substance use among the youth [14,15].

An additional questionnaire was used to gather data on other aspects of drug use. It contained three questions:

1. Who introduced you to drug use? Possible responses for this question were: a) A friend; b) A parent; c) A sibling; d) Other relative; e) Other (specify).

2. Which reason(s) explain why you use the substance you use? Possible responses for this question were: a) To relax; b) To relieve stress; c) To be accepted by peers (peer pressure); d) Excess pocket money; e) Desire to experiment; f) They are easily available; g) To enhance academic performance; g) To cope with problems; h) Other (specify).

3. What problems have you had that you attribute to your use of the substance(s)? Possible responses for this question were: a) Quarrel or argument; b) Scuffle or fight; c) Accident or injury; d) Loss of money or other valuable items; e) Damage to objects or clothing; f) Problems in your relationships with your parents; g) Problems in your relationship with your friends; h) Problems in your relationship with teachers; i) Performed poorly at school or work; j) Victimized by robbery or theft; k) Trouble with police; l) Hospitalised or admitted to an emergency room; m) Engaged in sex you regretted the next day; n) Engaged in unprotected sex; o) Blackouts or flashbacks; p) Medical problems e.g. memory loss, hepatitis, head injury, bleeding etc; q) Other (specify).

Both questionnaires were administered in the English language, given that this is the main language of instruction at secondary and tertiary educational institutions in Kenya.

Lifetime substance use in this study refers to respondents who admitted to having ever used at least one of the substances listed in the questionnaire.

### Procedure

The study was carried out in the period between May and September 2009. Ethical review was conducted by the Institutional Research and Ethics Committee of Moi University and Moi Teaching and Referral Hospital, and administrative approvals were sought and granted by the Uasin Gishu District Education Officer and the heads of the respective institutions. Questionnaires were given to the selected participants by members of the research team and collected on the same day after being completed. No names or identifying information were indicated on the questionnaires, and all participants were assured of absolute confidentiality.

### Data storage and analysis

Collected data was entered into a Microsoft Excel database and analysed using SPSS version 12.0. Descriptive statistics were used to compute means and standard deviations for numerical variables as well as frequencies for nominal and ordinal variables. The Chi square test statistic (χ^2^) was used to test the significance of association between various factors and the substance use measures, while the t-test was used to compare means. A value of *p *< 0.05 was considered statistically significant.

## Results

### Socio-demographic characteristics

Among the respondents, 261 (52.2%) were male and the mean age was 22.9 years (18-32, s.d. 2.5). Only 13.1% were married, and majority (59.7%) were staying at the institutional hostels. A minority of the respondents reported being employed, with jobs identified including business, accountancy, hairdressing and teaching. A majority of them (56.5%) depended on a government loan programme for higher education. Table 1 shows the socio-demographic characteristics of the study population.

**Table 1:** Socio-Demographic Variables by Gender

VARIABLE	MALE (%)	FEMALE (%)	TOTAL (%)
**Housing type**			
Hostel	131 (51.4)	159 (68.8)	290 (59.7)
Non-hostel	124 (48.6)	72 (31.2)	196 (40.3)

**Working**			
Yes	226 (90)	210 (91.7)	436 (90.8)
No	25 (10)	19 (8.3)	44 (9.2)

**Residence setting**			
Rural	76 (29.5)	52 (22.1)	128 (26)
Urban	182 (70.5)	183 (77.9)	365 (74)

**Marital status**			
Married	43 (16.9)	21 (9)	64 (13.1)
Single	212 (83.1)	212 (91)	424 (86.9)

**Government Loan**			
Yes	151 (59.7)	122 (53)	273 (56.5)
No	102 (40.3)	108 (47)	210 (43.5)

### Lifetime substance use

Among the participants, 349 (69.8%) reported having used at least one substance in their lifetime. Table 2 shows the association of lifetime substance use with selected socio-demographic variables.

**Table 2:** Lifetime Substance use Variation with Socio-Demographic Variables

VARIABLE	Lifetime substance use %	**X**^**2**^	p value
**Gender**			
Male	72.8	2.327	0.144
Female	66.5		

**Housing type**			
Hostel	68.3	0.744	0.421
Non-hostel	71.9		

**Working**			
Yes	70.5	0.015	0.863
No	71.3		

**Residence setting**			
Rural	70.3	0.001	1.000
Urban	70.1		

**Marital status**			
Married	70.3	0.000	1.000
Single	70.3		

**Government Loan**			
Yes	70.3	0.001	1.000
No	70.5		

There was no statistically significant association between lifetime prevalence of substance use and any of the selected socio-demographic variables.

### Specific Substance use

Among the 478 participants who answered the questions on alcohol use, 248 (51.9%) reported lifetime alcohol use in this study. Of these, 97.6% had had a drink in the preceding week, translating to a current alcohol use prevalence rate of 50.7% in this sample. There was no statistically significant variation of alcohol use with gender or any of the other socio-demographic variables. Table 3 shows the relationship between alcohol use and these variables.

**Table 3:** Lifetime Alcohol use Variation with Socio-Demographic Variables

VARIABLE	Lifetime Alcohol use %	**X**^**2**^	P value
**Gender**			
Male	53.6	0.609	0.464
Female	50.0		

**Housing type**			
Hostel	51.1	0.012	0.925
Non-hostel	51.6		

**Working**			
Yes	51.2	0.004	1.000
No	51.7		

**Residence setting**			
Rural	51.6	0.000	1.000
Urban	51.6		

**Marital status**			
Married	55.9	0.586	0.488
Single	50.6		

**Government Loan**			
Yes	50.4	0.251	0.642
No	52.7		

The average age at which the respondents started using alcohol was 17.5 years (11-23, s.d. 2.0), with males starting at 17.8 years (11-22, s.d. 2.0) and females starting at 17.3 years (12-23, s.d. 2.0). There was no statistically significant difference between male and female mean age of onset of alcohol use (t = 1.909, p = 0.06).

Over the month preceding the study, the mean number of days of alcohol use was 6.1 (1-20, s.d. 3.9). Respondents living in hostels drank on more days than those that were living outside college hostels (t = 3.058, p = 0.002). Those receiving government assistance also drank on more days than those not on government assistance programs (t = 3.516, p = 0.001). There was no statistically significant association between the mean number of drinking days per month and the other socio-demographic variables.

The mean number of drinks per drinking day over the preceding month was 2.9 (0-9, s.d. 1.8). As shown in Table 4, those living in hostels (t = 2.362, p = 0.019), those residing in urban areas (t = 3.203, p = 0.002) and those receiving government loan (t = 4.564, p = 0.000) reported statistically significantly higher rates than respondents without those attributes.

**Table 4:** Variation of Drinking Pattern with Socio-Demographic Variables

VARIABLE	Drinking days in the last month (Mean = 6.1 ± 3.9)	Mean number of drinks per drinking day (Mean = 2.9 ± 1.8)
**Gender**	t = 0.125, p = 0.901	t = -1.179, p = 0.239
Male	6.1 ± 3.7	2.8 ± 1.6
Female	6.1 ± 4.2	3.1 ± 2.1

**Housing type**	t = 3.058, p = 0.002*	t = 2.362, p = 0.019*
Hostel	6.8 ± 4.0	3.2 ± 2.0
Non-hostel	5.3 ± 3.7	2.6 ± 1.6

**Working**	t = -1.023, p = 0.307	t = -0.519, p = 0.604
Yes	7.0 ± 4.6	3.2 ± 1.4
No	6.1 ± 3.9	3.0 ± 1.9

**Residence setting**	t = -1.921, p = 0.056	t = -3.203, p = 0.002*
Rural	5.3 ± 4.0	2.3 ± 1.3
Urban	6.4 ± 3.9	3.2 ± 1.9

**Marital status**	t = 0.820, p = 0.413	t = -0.092, p = 0.927
Married	6.7 ± 4.2	2.9 ± 1.5
Single	6.1 ± 3.9	3.0 ± 1.9

**Government Loan**	t = 3.516, p = 0.001*	t = 4.564, p = 0.000*
Yes	7.0 ± 4.0	3.4 ± 2.1
No	5.2 ± 3.7	2.4 ± 1.2

*Statistically significant

Table 4 summarises the variation of drinking patterns with various socio-demographic variables.

Among the respondents using alcohol, 50.4% admitted having taken 5 or more drinks per day on one or two days in the preceding month, while 9.2% had done so on 3 or more days. The rest reported taking less than five drinks on each drinking day.

A total of 203 (42.8%) of the 474 participants who answered the question on smoking admitted to having smoked a cigarette at least once in their lifetime. The lifetime cigarette smoking prevalence rate was statistically significantly higher for the males than for the females (47.6% vs. 37.5%, X^2 ^= 4.922, p = 0.032). There was no statistically significant association between prevalence rate of cigarette smoking and any of the other variables.

Table 5 shows the relationship between cigarette smoking and various variables.

**Table 5:** Lifetime Cigarette use

VARIABLE	Lifetime Cigarette use %	**X**^**2**^	P value
**Gender**			
Male	47.6	4.922	0.032*
Female	37.5		

**Housing type**			
Hostel	43.1	0.018	0.925
Non-hostel	42.5		

**Working**			
Yes	45.2	0.081	0.871
No	43.0		

**Residence setting**			
Rural	40.5	0.418	0.530
Urban	43.8		

**Marital status**			
Married	41.4	0.059	0.888
Single	43.1		

**Government Loan**			
Yes	44.3	0.663	0.453
No	40.6		

*Statistically significant

The mean age at first cigarette smoking was 15.7 years (10-21, s.d 1.8). Females started smoking at the mean age of 16.0 years (s.d. 1.8) while males started at the age of 15.5 years (s.d. 1.7). This difference was statistically significant (t = -2.151, p = 0.033).

When asked how many cigarettes a day they had smoked 'in the past 30 days or month', 62.5% admitted to smoking 'less than one cigarette per day', while 25.6% smoked 1-5 cigarettes per day. A small proportion (4.4%) smoked 6-15 cigarettes a day, while 7.5% smoked 16 or more cigarettes each day.

Ten (2%) respondents admitted to using cannabis. Of these, 8 were male and 2 were female. Out of these, 6 admitted to having used cannabis in the past 12 months. Only 3 respondents admitted to ever having used cocaine.

### Who introduced respondent to substance use

Among the 349 participants who admitted to having ever used any of the above substances, 75.1% were introduced by a friend while 23.5% were introduced by a relative other than the nuclear family. A very small percentage of respondents in this sample (1.4%) were introduced to drug use by a member of the nuclear family.

### Reasons for substance use

Reasons given for substance use were: To relax (62.2%), to relieve stress (60.8%), desire to experiment (41.9%), peer pressure (38.9%), and to cope with problems (38.9%).

### Negative effects attributed to substance use

Most of the negative effects attributed to substance use by the respondents were associated with alcohol use. Significantly, 55.2% of the participants using alcohol reported having experienced medical problems as a result of their alcohol use. Further, 60.5% engaged in unprotected sex and 62.5% engaged in sex they regretted the next day. Over 60% of the participants reported engaging in scuffles, loss and damage to property and quarrels. Table 6 shows the negative effects attributed to the use of alcohol.

**Table 6:** Negative Effects Attributed to Alcohol Use

PROBLEM	% FREQUENCY (N = 248)
Quarrel/argument	82.7
Damage to objects	68.5
Problem in relationship with parents	68.1
Scuffle/Fight	68.1
Loss of money or other valuable item	68.1
Accidental injury	63.3
Engaged in sex you regretted the next day	62.5
Engaged in unprotected sex	60.5
Medical problems	55.2
Robbery or Theft	52.3
Problem in relationship with friends	52.0
Trouble with police	50.8
Poor performance at school	42.3
Problems with teachers	41.5

Among cigarette smokers, the problems they attributed to cigarette smoking were in relationships with teachers (20.7%), parents (11.8%) and friends (6.9%). Some of the respondents attributed medical problems (8.4%) and poor school performance (4.4%) to cigarette smoking. None of the respondents attributed any problems to substance use other than alcohol or cigarette use.

## Discussion

This study found a lifetime substance use prevalence rate (69.8%) that is significantly higher than the 41% rate found among high school students in Kenya [2]. This implies that substance use rates, in general, increase with age and transition through the education system. This has major policy implications, including the need to focus substance use interventions on younger age-groups such as primary and high school students. Preventing early substance-related problems will reduce the risk of these problems in later adulthood when the magnitude of life stresses is greater.

The prevalence rate of lifetime alcohol use in this study was 51.9%. This rate, though lower than what others have found in similar settings, is still significant. Odek-Ogunde et al [5] found rates of lifetime alcohol use as high as 84% among students at a private university in Nairobi, Kenya, while Othieno et al [16] in a more heterogeneous population consisting of outpatients attending several Nairobi primary health care facilities found a lifetime alcohol use prevalence rate of 62%.

The paucity of literature on alcohol use among college students in Africa and other parts of the world was demonstrated by Karama et al [17] in a 2006 review article in which the authors argued for the need for more studies to be carried out in this area in order to inform interventions. However, studies among high school students in this region report similarly high prevalence rates of alcohol use, ranging from 15% to 57.9% [2,4,6].

Almost all (97.6%) of those who had ever used alcohol in this study could be regarded as current users (had an alcoholic drink in the week preceding the study), translating to a 50.7% current alcohol use prevalence rate. In contrast, a Kenyan general population study [18] found a current alcohol use prevalence rate of 13.4%. The huge difference implies that alcohol use is probably still regarded as being fashionable among college students and very few social sanctions exist to discourage this behaviour. Chances of moving from use to abuse and dependence are therefore heightened, especially considering other factors such as age of onset and frequency of use.

Participants reported having used alcohol on an average of 6 days in the month preceding the study. The only factors significantly associated with this were housing type and government loan. Students residing in hostels drank on average 1.5 days more than those not residing in hostels, suggesting a significant role for peer influence in shaping drinking behaviour. Participants receiving government loan reported drinking almost 2 days more than those not receiving such loans, probably reflecting more readily available funds to sustain drinking behaviour. However, other factors may be responsible for this finding, and further research would be necessary to clarify this. Students in tertiary institutions in Kenya may apply for a Higher Education Loan from the government, and many of them are loaned various amounts of money depending on the course of study and the institution. The loan is recovered once the loanee completes their studies and secures employment or a steady source of income.

The mean number of days the participants reported drinking suggests that they drink between one and two days each week, pointing towards a binge-drinking pattern rather than regular daily intake. In further support for the likelihood of a binge-drinking pattern of alcohol use, almost 60% of the participants in this study reported taking 5 or more drinks per day at least once in the preceding month.

Previous research suggests that although it is difficult to generalise alcohol use patterns in many African countries, binge-drinking is a common phenomenon in the Kenyan general and even clinical population. For instance, a study by Saunders et al among patients at primary health care centres [19] reported that out of the six countries involved, the Kenyan sample had the highest rates of binge-drinking (episodic heavy drinking). The other countries in the study were the USA, Mexico, Australia, Norway and Bulgaria.

A community based survey by Clausen et al [18] reported that 27.6% of the Kenyan current drinkers had had 5 or more drinks on at least one occasion in the preceding week, suggesting that binge-drinking was a common phenomenon in the Kenyan drinking population. This pattern of drinking would also explain the high prevalence of reported serious problems associated with alcohol use in our study, such as scuffles, loss and damage to property, medical problems and sexual indiscretions.

An average of 3 drinks per drinking day was found among alcohol users in this study, with students living in hostels, in urban areas and receiving government loan taking more drinks on average than those without those attributes. These findings further illustrate the role of peer influence and urbanisation on alcohol use. The possibility of under-reporting cannot be discounted, given that the participants in this study were students who may be reluctant to reveal their drinking habits, even on an anonymised questionnaire.

In the study by Saunders et al [19] which was carried out in a primary health care setting, the mean daily number of drinks taken by the 'drinking population' was 9.6, while those classified as alcoholics were taking a mean of 23.4 drinks. Another study in a general population setting [18] had found a mean weekly drink rate of 9.9 drinks among Kenyan regular alcohol users, many of whom displayed a binge-drinking pattern of alcohol use.

In this study, the mean age at first alcoholic drink was 17.5 years, with the youngest reported age being 11 years. There was no statistically significant difference in male and female age of onset of alcohol use. Otieno and Ofulla [6] similarly found the highest prevalence of alcohol use among young people aged 16-18 years. Several other studies have reported early age of onset of alcohol use among adolescents and the associated psychological problems in later life [9,20,21].

Despite the fact that the legal drinking age in Kenya has been set at 18 years for a long time, it is clear that alcohol is available for sale even to underage drinkers. As has been observed before [22], failure of enforcement may be part of the problem, as well as the domination of the alcohol market by small-scale often illicit producers and sellers. Partanen [22] suggests that 80-90% of total alcohol consumed in Kenya comes from 'small-scale production within the informal sector, licit or illicit, using traditional African methods of brewing and local skills of distilling'.

Among some of the problems associated with early age of onset of alcohol use is increased risk of later alcohol abuse and dependence as well as associated social and occupational difficulties [20,21]. In one study, Grant and Dawson showed that the odds for dependence decreased by 14% while those for abuse decreased by 8% with each increasing year of onset of use [20]. The implication of the high rate of alcohol use and early age of onset in this study is that a large proportion of the respondents are at a high risk of developing alcohol-related disorders as adults.

In the present study, negative effects attributed to alcohol use by the respondents included quarrels and fights, loss and damage to property, regretted sex, unprotected sex, and medical problems. As indicated earlier, most of these problems could be attributed to a binge-drinking pattern of behaviour, rather than regular light use of alcohol. The high risk sexual behaviour is particularly ominous due to the high prevalence of HIV and other sexually transmitted infections in Kenya.

In 2007, Chersich et al [23] reported an association between binge-drinking and unsafe sex, sexual violence and sexually transmitted infections among Kenyan female sex workers, and the students in the present study are clearly exposed to the same risks. It is clear that unless substance use among adolescents and young adults is addressed, interventions targeting HIV/AIDS, violence and accidents will achieve less than optimal results.

The lifetime prevalence rate of cigarette smoking in this sample was 42.8%, which is higher than what has been reported elsewhere [3,4,6,8]. However, most of these prior reports focused on adolescents in high school, once again raising the possibility that the rates of cigarette use increase with age and academic progression.

In this study, there was a statistically significant difference in the lifetime cigarette use prevalence rates between males and females, with males having a higher rate than females. This is consistent with what has been found in other studies, and may reflect a more tolerant social attitude to male than to female smoking [2-5,16,24].

The mean age at first cigarette use in this study was 15.7 years, and the youngest reported age was 10 years. Males had a significantly lower mean age of onset of cigarette use than females. The Kenyan Tobacco Control Act (2007) prescribes the minimum smoking age to be 18 years, yet tobacco products appear to be available to people as young as 10 years old. The same regulatory problems identified in relation to alcohol use are probably also operational with regard to tobacco use in Kenya.

Elsewhere, Kwamanga et al [3] reported an age of cigarette smoking onset of 12 to 16 years, while Peltzer [24] reports a mean age of onset of 14.8 years, similar to the findings in this study. Early onset of cigarette use has obvious implications for these young people in social and academic spheres. As a matter of fact, the commonest smoking-attributed problems reported by participants in this study were associated with teachers, parents and physical health. Although it was beyond the scope of this study to identify exactly what sort of problems the smokers encountered with teachers and parents, it may be presumed that they were probably related to discipline and interpersonal conflict. Smoking is outlawed in learning institutions in Kenya, as well as in most public places.

Although only a small proportion (7%) of those who smoked reported using more than 16 cigarettes a day, this is still significant in view of the health risks posed by this amount of exposure to cigarette smoke. It also suggests that this group is already dependent on nicotine, and this may cause problems in important areas of functioning including school, social and family functioning.

The rates of other substance use in this study were low. This may be attributed to either under-reporting or a lack of availability of these substances. It is difficult to ascertain the true reason for the low prevalence rates since the questionnaire used was a self-report format and there was no follow-up of responses. However, this finding appears consistent with the previous studies in the same environment, where reports of illicit drug use have been low [2,5,16]. Only cannabis use has been found to be more common in the earlier studies compared to the present one. For instance, Odek-Ogunde [5] reported a 19.7% lifetime prevalence rate of cannabis use among university students, but found rates less than 5% for drugs such as heroin, cocaine and amphetamines.

Majority of those reporting lifetime substance use indicated that they were influenced by a friend or relatives other than the nuclear family. Similar findings have been reported in other studies, confirming the role of peer pressure and social learning in initiation of substance use [8,24]. The implication of this finding is that peers and older relatives would also serve as good role models for a substance use intervention program for young people in this setting.

In this study, most respondents indicated that they used substances to relax and relieve stress. Madu and Matla found that majority of drug using adolescents do so when they are bored, tired or stressed up, or at parties [4]. A 1987 Medical Student survey in the US also found similar reasons for most of the drugs used, including to relax, to have a good time, to feel good and even to experiment [1]. This raises the possibility that interventions that help young people to use their time more productively would reduce the incidence of substance use.

This study encountered a number of limitations, chief among them being the relatively small sample size and a study design that precludes generalisation of the results to students in other institutions. Due to the heterogeneity of the institutions and the courses offered in this region, it would take a larger study to fully describe the substance use patterns among college students in Kenya or even within Eldoret Municipality. The information generated from this study may however be useful in the design of such a study.

Another key limitation is the fact that a self-administered questionnaire was used to collect data on current and lifetime substance use. Without any other validating measures it is not possible to conclude with certainty that the information generated is an accurate representation of substance use in this population. However, the WHO model questionnaire has been used elsewhere [2,14] under similar circumstances, and therefore the results are at least comparable to those found in similar studies.

## Conclusions

This study has demonstrated a high prevalence of substance use among college and university students in a low income country, with the most commonly used substances being alcohol and cigarettes. Very serious effects have also been reported, with a significant proportion of those using alcohol attributing serious physical and psychosocial effects to its use. The risk of substance dependence and other mental and general medical conditions is therefore very high in this population, and targeted interventions are recommended to deal with this issue expeditiously. Due to the early age of onset of substance use found in this study, it is recommended that these interventions must target people as young as 10-12 years, and involvement of peers and role models would have a high probability of success.

## Competing interests

The authors declare that they have no competing interests.

## Author contributions

LA was involved in the conceptualisation of the study idea, development of the study design and instruments, supervision of data collection and analysis, and preparation of the final manuscript. PAM, MNN, KCK and EMO were involved in conceptualisation of the study idea, design of the study, data collection and analysis. All authors were involved in the approval of the final manuscript.

## Pre-publication history

The pre-publication history for this paper can be accessed here:

http://www.biomedcentral.com/1471-244X/11/34/prepub
